# Waterpipe smoking cessation: knowledge, barriers, and practices of primary care physicians- a questionnaire-based cross-sectional study

**DOI:** 10.1186/s12875-020-1095-4

**Published:** 2020-01-30

**Authors:** Maya Romani, Sarah Jawhar, Manar Shalak, Jumana Antoun

**Affiliations:** Department of Family Medicine, American University of Beirut, Riad El Solh, Beirut, 11-0236 Lebanon

**Keywords:** Waterpipe smoking, Smoking cessation, Family physicians

## Abstract

**Background:**

While cigarette smoking has been considered the most relevant tobacco product worldwide, waterpipe tobacco smoking (WTS) has increased in prevalence globally and calls for more considerable attention now. However, little is known about WTS cessation knowledge and clinical practices among physicians, particularly in Lebanon. This study aims to examine the knowledge, barriers, and cessation practices of primary care practitioners towards WTS.

**Methods:**

A cross-sectional study where an anonymous self-reported questionnaire was completed by physicians attending the Annual Conference of the Lebanese Society of Family Medicine for family medicine physicians, general practitioners, and internists in Lebanon.

**Results:**

Out of 180 attendees, 105 primary care practitioners (PCPs) responded to the questionnaire. Only 38.1% of the physicians think similar techniques are used for the cessation of smoking of both cigarette and waterpipe. Similarly, 30.5% of the physicians believe that nicotine replacement therapy works in the cessation of waterpipe smoking. There was a statistically significant difference between the percentage of physicians who counsel for cigarette smoking and those who counsel for waterpipe smoking cessation (*p* = 0.005) where 30% of the physicians tend to counsel against cigarette smoking more than waterpipe smoking.

**Conclusions:**

This study shows a difference in the attitude and behavior of PCPs towards cigarette and waterpipe smoking cessation. Moreover, there is a lack of knowledge about water pipe smoking cessation techniques. There is a great room for continued medical education to PCPs in their private practice to improve their knowledge.

## Background

Since the turn of the twenty-first century, the tobacco product landscape has witnessed the emergence of a new wave of tobacco products, including waterpipe tobacco smoking (WTS), which has triggered alarming epidemiological trends [1, 2]. While this practice (also known as *arghile, hookah, and shisha*) was limited to Southeast Asia and Eastern Mediterranean communities, it has recently begun to rise in popularity in other regions of the world [3, 4]. Both waterpipe tobacco users and non-users perceive WTS to be less harmful than cigarette smoking [5, 6]. However, there exists compelling evidence about the harms of waterpipe smoking and detrimental effect on long-term health outcomes [7, 8]. More specifically, several systematic reviews and meta-analyses have demonstrated the association between WTS and increased risk of lung cancer, cardiovascular diseases, abnormal pulmonary function, low birth weight, and periodontal diseases [4, 9–11]. Despite the evidence, the incorrect public perception of reduced harm is possibly driven by the introduction of flavored waterpipe tobacco (also known as Maassel), the advancement of global communication particularly the internet, and the lack of enforcement of waterpipe tobacco control policies [6, 12].

Given the growing rate of the waterpipe epidemic specifically among young adults, a recent meta-analysis has reported the Eastern Mediterranean Region to have the highest estimates for ever use of waterpipe tobacco among youth ranging from 12.9% among secondary school in Iran to 65.9% among secondary school and university students in Lebanon [5, 13]. Meanwhile, data from other regions of the world demonstrate a similar trend. For example, recent findings from the National Adult Tobacco Survey conducted in the USA involving 118, 581 participants showed that young adults (18–24 years) had the highest prevalence rate of WTS (28.6%) compared to older adults [14]. Similarly, in Canada, the national Youth Smoking Survey that was administered in 2012/2013 revealed that 14.3% of Canadian students in grades 9–12 (*N* = 27,404) have reported to ever using waterpipe [15].

Therefore, smoking cessation and its associated health benefits are crucial in treating tobacco dependence through a medical intervention that includes pharmacological and behavioral approaches [16]. In addition, physicians play a significant role in promoting smoking cessation among patients [17]. More specifically, primary care physicians (PCPs) have a significant opportunity in reducing smoking rates through effective counseling since they serve as the first point of medical contact for patients, including smokers [17–19]. However, the literature shows that several factors and barriers influence physicians’ provision of smoking cessation advice. Some of the identified barriers include lack of awareness, insufficient time, lack of self-efficacy, and lack of training [20, 21].

Consequently, there is an urgent need for WTS to be better understood among healthcare professionals, especially among PCPs, to improve the delivery of tobacco cessation interventions [22]. Effective smoking cessation strategies and barriers to the provision of assistance among physicians had been comprehensively studied previously. Still, rare studies have examined the knowledge and evidence-based cessation clinical practices among physicians in relation to waterpipe tobacco use [23, 24].

Therefore, the purpose of this study was to explore the knowledge, attitude, practices, and barriers associated with waterpipe smoking among Lebanese PCPs. We aimed to (i) assess knowledge and attitudes regarding waterpipe smoking among physicians; (ii) assess clinic-based practices related to cigarette vs. waterpipe smoking cessation among these physicians, and (iii) assess providers’ perceived barriers to providing WTS cessation intervention.

## Methods

### Study design

This was a cross-sectional study assessing the knowledge, attitude, and barriers towards waterpipe smoking cessation clinical-based practices among Lebanese family medicine physicians. Data was collected from self-reported questionnaires completed by physicians who attended the Annual Conference of the Lebanese Society of Family Medicine. The conference is a well-respected conference and is highly attended by family medicine physicians (approximately one-third of the total registered family physicians in the country). In Lebanon, there are around 250 registered family physicians; however, many of them practice abroad. The study received approval from the American University of Beirut Medical Center’s (AUBMC) Institutional Review Board.

### Study population

The study population included a convenience sample of the conference attendees (180PCPs). The attendees comprised of family medicine doctors, general practitioners, and internists residing and practicing family medicine in Lebanon.

### Instrument development

A questionnaire was developed in English by family medicine physicians at AUBMC and consisted of 21 questions covering the following topics (See Additional file 1):

1. Physicians’ demographic information and tobacco use status
2. Physicians’ knowledge and attitudes’ towards WTS

- Familiarity with the waterpipe apparatus and its toxic content
- Familiarity with the health outcomes of WTS (dependence, addiction, and diseases)

3. Physicians’ smoking cessation clinical practice

- Practicing the USPHS Clinical Practice Guidelines (Using the 5 A’s)
- Frequency of counseling patients on cigarette smoking and WTS

4. Physicians’ barriers to the provision of WTS cessation assistance

- Level of importance of perceived barriers to counseling patients on waterpipe smoking cessation

### Data collection

Upon arrival at the conference and after completing registration, in-person oral consent was obtained from the attendees who were informed that the questionnaire was anonymous and voluntary. The questionnaires were distributed to the participants while the conference was taking place. After filling out the questionnaires, attendees dropped the forms in a concealed box localized next to the registration area.

### Data analysis

Data were analyzed using SPSS statistical software IBM 19. Frequencies and percentages were used to measure the knowledge, practice, and barriers towards waterpipe smoking cessation. Chi-square and Fisher exact tests were used to compare the attitudes, knowledge, and barriers among the various demographics’ characteristics of PCPs. Statistical significance was defined as *p*-value ≤0.005.

## Results

### Physicians’ characteristics

Of the 180 PCPs who attended the conference, 105 physicians completed the questionnaire (response rate = 58%). The demographics and relevant characteristics of the respondents are shown in Table 1. Only 14.4% of participants were smokers, two thirds were family medicine physicians (63.1%), and almost all the physicians worked in Lebanon (98%). Less than half of the physicians had work experience of more than 15 years (43%) whereas half (54%) had a smoking cessation program at their practice. However, nearly two thirds (65.2%) had not received training in smoking cessation.

**Table 1:** Demographic and relevant characteristics of the PCPs

Characteristic	Total (*N* = 105)	
	N	%
Gender (N = 105)		
Males	60	57.1
Females	45	42.9
Specialty (N = 103)^a^		
Family Medicine	65	63.1
Internal Medicine	9	8.8
General Practitioners	28	27.1
Emergency medicine	1	1.0
Work experience (years) (N = 105)		
Less than 5 years	17	16.2
5–10 years	12	11.4
11–15 years	17	16.2
More than 15 years	45	42.9
Resident	14	13.3
Place of work^b^		
Academic Institution	52	49.5
Private Practice	62	59.0
Managed Care Organization	27	25.7
Country of work^a^ (N = 104)		
Lebanon	102	98.1
Bahrain	1	1.0
UAE	1	1.0
Received/participated in training program for smoking cessation^a^ (N = 95)		
Yes	33	34.7
No	62	65.2
Presence of smoking cessation programcessationprogram at the work of place*	57	54.3
Smoking status^a^ (N = 104)		
Smoker	15	14.4
Cigarettes	9	60
Waterpipe	5	33.3
Cigar	1	6.7
Non-smoker	86	82.7
Ex-smoker	3	2.9
Cigarettes	3	100

^a^Missing values exist

^b^More than one answer was allowed

### Knowledge and attitude of PCPs

The results of the questions asked to assess the participants’ knowledge regarding waterpipe smoking are summarized in Table 2. Most respondents answered correctly on three to four waterpipe statements (79%) while few answered one correctly (7%) or all incorrect (2%). Physicians were knowledgeable about the harmful and addictive ingredients in waterpipe smoking as well as the associated health risks, but almost half of the physicians were not aware of the cigarette equivalence of waterpipe smoking.

**Table 2:** Percent of PCPs giving correct answers on WTS statements

Statement	Agree	Neutral	Disagree
	N = 101 (%)		
Water pipe contains little toxicants because the smoke passes through a small receptacle of water.^a^	9 (9.0)	11 (11.0)	80 (80.0)
Water pipe delivers the addictive drug nicotine as is the case of cigarette.	80 (79.2)	12 (11.9)	9 (8.9)
During one session the water pipe smoker may inhale as much smoke as in 100 cigarettes.	54 (53.5)	17 (16.8)	30 (29.7)
Water pipe smokers are at risk for the same diseases as cigarettes smokers (cancer, heart and lung disease).	92 (91.1)	5 (5.0)	4 (4.0)

^a^Missing values exist (*N* = 100)

### Smoking cessation practices among PCPs

Nearly all physicians surveyed inquire about their patients’ smoking status (Table 3). Family medicine physicians ask their patients about the type of tobacco product smoked more often than internal medicine physicians and general practitioners (90.8% vs. 88.9% vs. 64.3%, *p* = 0.005). Similarly, physicians who worked at academic institutions were more likely to ask the patient about type of smoking than those who worked at non-academic workplaces (92.3% vs. 75.5%, *p* = 0.043).

**Table 3:** Smoking cessation practices of PCPs

Question	Often	Sometimes	Seldom/Never
	N = 105 (%)		
How often do you ask if your patient smokes?	99 (94.3)	3 (2.9)	3 (2.9)
How often do you ask your patients about type of smoking?	88 (83.8)	14 (13.3)	3 (2.9)
How often do you counsel your patients about cigarette smoking cessation?	79 (75.2)	20 (19.0)	6 (5.7)
How often do you arrange follow up visits to discuss cigarette smoking cessation?	17 (16.3)	42 (40.4)	45 (43.3)
How often do you warn your patients about waterpipe health dangers?	78 (75.0)	15 (14.4)	11 (10.6)
How often do you counsel patients about waterpipe smoking cessation?	65 (63.1)	22 (21.4)	16 (15.5)
How often do you arrange follow up visits to discuss waterpipe smoking cessation?	17 (16.5)	26 (25.2)	60 (58.3)

On examining intervention methods, 38.1% of physicians thought similar techniques are used for both cigarette and waterpipe smoking cessation. However, only 31.7% of respondents believe that nicotine replacement therapy (NRT) works in waterpipe smoking cessation. As for counseling, there was a statistically significant difference between those who counselled against cigarette smoking vs. waterpipe smoking. 12.4% of those who counsel against cigarette smoking do not counsel for WTS. This was paralleled by the result that 29.3% of the physicians answered that they tend to counsel against cigarette smoking more than waterpipe smoking and another 19.2% were neutral. Moreover, 80.2% of non-smokers physicians often counseled against cigarettes compared to 53.3% of smoker physicians (*p* = 0.048) (excluding 3 ex-smokers). However, no significant relation was found between counseling against WTS and physicians’ smoking status.

Meanwhile, there was a statistically significant difference between arranging for follow-up visits for cigarette smoking vs. waterpipe smoking; however, 29.3% of physicians who often/sometimes arrange for following for cigarettes fail to do that for those who smoke waterpipe (*p* = 0.000).

### Perceived barriers to providing waterpipe smoking cessation intervention

The three perceived barriers recognized by most physicians as very important to the provision of waterpipe smoking cessation advice were patient incompliance (79.1%), limited training and knowledge on smoking cessation (78.7%), and lack of available cessation programs (77.5%) (Table 4). These are followed by patients’ disinterest in quitting (72.5%) and lack of time during patient consultation (69.8%). Meanwhile, half (50%) of the respondents believed that the smoking status of a physician is not a significant barrier to counseling patients on waterpipe smoking cessation.

**Table 4:** Level of importance of perceived barriers to the provision of waterpipe smoking cessation intervention among PCPs

Perceived barrier	Very important/important	Neutral	Not important
	N (%)		
Patients are not compliant	72 (79.1)	5 (5.5)	14 (15.4)
Limited training and knowledge on smoking cessation	60 (78.7)	6 (6.7)	13 (14.6)
Lack of available smoking cessation referring clinic or program	69 (77.5)	11 (12.4)	9 (10.1)
Patients are not interested	66 (72.5)	12 (3.2)	13 (14.3)
Lack of time during patient consultation	60 (69.8)	18 (20.9)	8 (9.3)
Patients lack awareness about water pipe harms	63 (69.2)	9 (9.9)	19 (20.9)
Lack of personal knowledge about water pipe smoking cessation	61 (67.8)	12 (13.3)	17 (18.9)
Waterpipe smoking is considered a norm in special places by the community	57 (63.3)	11 (12.2)	22 (24.4)
Lack of knowledge about the harms of waterpipe	54 (60.0)	11 (12.2)	25 (27.8)
Cost of medications and clinic visit	53 (58.9)	21 (23.3)	16 (17.8)
No expected benefit as patients will continue to smoke anyway	45 (52.3)	20 (23.3)	21 (24.4)
The physician smokes	29 (38.2)	9 (11.8)	38 (50.0)

## Discussion

### Summary

Implementing effective evidence-based tobacco control strategies in Lebanon is crucial in combating the expanding tobacco epidemic. Unfortunately, clinical smoking cessation guidelines have focused on traditional cigarette consumption with a limited concentration on alternative tobacco products such as WTS, which calls for urgent acknowledgement and understanding and WTS cessation approaches among PCPs who play a vital role in facilitating smoking cessation [25].

### Strengths and limitations

This is the first study to investigate the knowledge, barriers, and clinical-based practices of Lebanese PCPs’ regarding WTS smoking cessation interventions. To our knowledge, there are no studies of a similar subject in Lebanon or worldwide to make comparison. This is the main strength of this study. The relatively small sample in this study might make it difficult to assess the causation of the lack of information regarding the waterpipe smoking cessation practice. As the participants were recruited at a medical conference, it may affect generalizability to primary care physicians in Lebanon. Nevertheless, the fact that the results showed a lack of knowledge about waterpipe smoking cessation techniques as compared to cigarette smoking is alarming, taking into consideration that the sample is supposed to be more up-to-date in medical knowledge as they are conference attendees.

### Comparison with existing literature

#### Demographic characteristics

In this study, only 14.3% of the respondents were smokers, which is less than the national smoking rate (38.5%). This rate is lower than in other studies carried out in Makkah, Saudi Arabia among primary health-care physicians (26%) and in Alexandria, Egypt among primary health care (PHC) personnel (45%) [26, 27]. However, this figure is higher compared to studies done in developed countries such as USA (7%) and UK (4%).

While 54% reported having a smoking cessation program at their practice, only 34.7% of PCPs reported receiving or participating in a training program for smoking cessation which is higher than that found in Egypt (30%), Jordan (26.6%), Bahrain (11.6%), Kuwait (12.6%), and Armenia (26.9%) [3]. However, this result was lower than that found in Palestine (43.2%) and Riyadh (68%) [28, 29]. These responses might explain why 78.7% believed that limited training and knowledge in this field is perceived as a very significant barrier to providing smoking cessation assistance to patients. One way to eliminate the barrier associated with lack of training is by training physicians in smoking cessation, during medical school or in the form of continuing education, since it has been positively associated with better practice and engagement in smoking cessation according to studies performed in Egypt and Switzerland [27, 30, 31].

#### Knowledge and attitudes

The present study revealed existing knowledge among most physicians (79%) about the harms of waterpipe smoking, its associated risk of dependence, and its detrimental health outcomes. This is way higher than a study done in England where it shows that over half of general practitioners were knowledgeable on the topic [32]. However, this finding is in accordance with data from a study conducted among physicians regarding shisha smoking in Pakistan. In addition, half of our respondents were not familiar with WTS and its cigarette equivalence, similar to a study done in England [32]. One reason could be due to a lack of awareness among physicians about research regarding the lethal toxins and chemicals added to waterpipe smoke. According to the literature, waterpipe smoke contains higher levels of metals such as arsenic, lead and nickel, 36 times more tar, 15 times more carbon monoxide, and up to 10 cigarettes’ worth of nicotine and 100 cigarettes’ worth of smoking inhalation for a single waterpipe use episode.

#### Smoking cessation practices

Most surveyed physicians often inquired about their patients’ smoking status and tobacco use (94.3 and 83.8%, respectively). This rate is higher than that found in Egypt (49.5%), Saudi Arabia (73.4%), and the U.S (66.4%), which suggests that almost all our PCPs follow the U.S public health service clinical practice guidelines regarding tobacco dependence [28, 30]. This has a tremendous effect on tobacco smoking cessation since brief advice (≤ 5 min) in clinical settings can lead to a quit rate of 2 to 4%, and PCPs are the first to hold this responsibility.

In our study, family medicine physicians asked and counseled their patients about the type of tobacco product smoked more than internal medicine physicians and general practitioners (90.8% vs. 88.9% vs. 64.3%, *p* = 0.005). Similarly, a national study in the U.S. revealed that PCPs were more likely than other specialties to offer smokers assistance to quit, however, regarding asking their patients about their smoking status, all healthcare professionals, including primary care physicians, psychiatrists, and emergency physicians) do that equally.

Regarding the intervention methods, 38.1% thought similar techniques are used for both cigarette and waterpipe smoking cessation. But still physicians tend to counsel against cigarette smoking more than against WTS (29.3%). Moreover, 29.3% of physicians who arrange for follow-up for cigarette fail to do so for waterpipe. This indicates the lack of evidence on interventions and lack of knowledge.

#### Physicians’ perceived barriers to providing WTS cessation interventions

The majority of the physicians believed that patient incompliance, limited training and knowledge on smoking cessation, lack of available cessation programs, patients’ disinterest in quitting and lack of time during patient consultation are very important barriers that interfere with promoting waterpipe smoking cessation. Our findings are consistent with earlier studies conducted in Gulf countries and Europe that have examined factors influencing PCPs’ engagement in smoking cessation [9, 17, 20, 21], which have revealed time constraint [21, 33–35], lack of training [21, 28, 33–35], and patients’ disinterest in quitting [28] to be the most common perceived barriers among healthcare professionals. Interestingly, 50% of the physicians did not believe that their own smoking status is an important barrier that affects their smoking cessation practices which is in accordance with studies performed in Palestine, Kuwait, and Bahrain where respondents were in the lowest agreement with the statement. However, these studies investigated barriers addressing smoking cessation targeting cigarette consumption, but not waterpipe smoking cessation.

## Conclusions

This study shows a difference in the attitude and behavior of PCPs towards cigarette and waterpipe smoking cessation. Moreover, there is a lack of knowledge about waterpipe smoking cessation techniques. This is due to the lack of training for physicians. Hence, eliminating this barrier by introducing smoking cessation techniques, early on in the physicians’ medical education in medical school is crucial. In addition, there is a great room for continued medical education to PCPs in their private practice to improve their knowledge.

## Supplementary information

**Additional file 1.** Family physicians and waterpipe smoking cessation: survey of attitudes and barriers

## Data Availability

All data generated or analysed during this study are included in this published article.

## Abbreviations

NRT: Nicotine replacement therapy
PCPs: Primary care practitioners
PHC: Primary health care
USPHS: United States public health service
WTS: Waterpipe tobacco smoking
