# Supplementary material for: Waterpipe smoking cessation: knowledge, barriers, and practices of primary care physicians- a questionnaire-based cross-sectional study
Source: BMC Fam Pract. 2020 Jan 30;21:21. doi: 10.1186/s12875-020-1095-4 (PMC6990485; doi:10.1186/s12875-020-1095-4)
Supplement: Supplementary file 1 — Additional file 1. Family physicians and waterpipe smoking cessation: survey of attitudes and barriers [file 12875_2020_1095_MOESM1_ESM.pdf]

Additional file 1

Questionnaire No: -------------

Family physicians and waterpipe smoking cessation: survey of attitudes and barriers

Please circle the right answer:

1- Gender: 1. Male 2. Female

2- Specialty: Please specify: ……………………………….

3- Years of work experience:
1. Less than 5 years 2. 5 to 10 years 3. 11-15 years 4. More than 15 years
5. Not applicable because I'm a resident

Place of work: (You can choose more than one)
1. Academic institution 2. Private Practice 3. Managed Care organizations
4. Others: _________________

4- Country of work: 1. Lebanon 2. Other, please specify:
5- Do you have smoking cessation program at your practice? 1. Yes 2. No

6- Smoking status: 1. Smoker (Pack year: ………….) 2. Nonsmoker 3. Ex-smoker

7- Type of smoking: (You can choose more than one)
1. Water pipe 2. Cigarettes 3. Cigar

8- How often do you ask if your patient smokes?
1. Often 2. Sometimes 3. Seldom 4. Never

9- How often do you ask your patients about the type of smoking?
1. Often 2. Sometimes 3. Seldom 4. Never

10- How often do you counsel your patients about **cigarette** smoking cessation?
1. Often 2. Sometimes 3. Seldom 4. Never

11- Do you use the 4 As (ask, advise, assist, arrange) to counsel patients who are not willing to quit **cigarette** smoking? 1. Yes 2. No

12- How often do you arrange follow up visit to discuss **cigarette** smoking cessation?
1. Often 2. Sometimes 3. Seldom 4. Never

13- How often do you warn your patients about **water pipe** health dangers?
2. Often 2. Sometimes 3. Seldom 4. Never

14- How often do you counsel your patients about **water pipe** smoking cessation in your practice?
1. Often 2. Sometimes 3. Seldom 4. Never

15- How often do you arrange follow up visit to discuss **water pipe** smoking cessation?
1. Often 2. Sometimes 3. Seldom 4. Never

16- Do you think similar techniques are used for cessation of smoking of both cigarette and water pipe?
1. Yes 2. Maybe 3. No 4. I don't know

17- Do you think that nicotine replacement therapy works in cessation of water pipe smoking?
1. Yes 2. Maybe 3. No 4. I don't know

18- In my practice I tend to counsel against cigarette smoking more than water pipe smoking?
1. Agree 2. Neutral 3. Disagree

19- Have you received or participated in training program for smoking cessation? 1. Yes 2. No

20- **Please indicate your level of agreement with the following statements (Please put X)**

**Statement**	**Agree**	**Neutral**	**Disagree**
Water pipe contains little toxicants because the smoke passes through a small receptacle of water			
Water pipe delivers the addictive drug nicotine as is the case of cigarette			
During one session the water pipe smoker may inhale as much smoke as in 100 cigarettes			
Water pipe smokers are at risk for the same diseases as cigarettes smokers (cancer, heart & lung disease, pregnancy adverse effects)			

21- What is the importance of the listed below barriers for not counseling your patients about **<u>water pipe</u>** smoking cessation in your practice?
1: very important 2: important 3: neutral 4: not important

**Barrier**	**Importance**
Patients are not compliant	
Patients are not interested	
No expected benefit, patients will continue to smoke anyway	
Waterpipe smoking is considered norm in social places by the community	
Patients' lack of awareness about water pipe harms	
Cost of medications and clinics visit	
Lack of adequate training and knowledge about smoking cessation	
Lack of personal knowledge about water pipe harms	
Lack of personal knowledge about water pipe smoking cessation techniques	
Shortage of time	
Lack of available smoking cessation referring clinic or program	
I smoke myself	
Others, please specify:____ ____	
